# Congenital tracheobronchomegaly (Mounier-Kuhn syndrome) in a 28-year-old Zambian male: a case report

**DOI:** 10.11604/pamj.2021.40.153.31703

**Published:** 2021-11-12

**Authors:** Saifurrahman Shahin, Thijs Hoffman, Wouter van Es, Jan Grutters, Kondwelani Mateyo

**Affiliations:** 1 Department of Internal Medicine, University Teaching Hospital, Lusaka, Zambia,; 2 Department of Pulmonology, St. Antonius Hospital, Nieuwegein, The Netherlands,; 3 Department of Radiology, St. Antonius Hospital, Nieuwegein, The Netherlands,; 4 Division of Heart and Lungs, University Medical Center, Utrecht, The Netherlands

**Keywords:** Tracheobronchomegaly, Mounier-Kuhn syndrome, Zambia, bronchiectasis, recurrent respiratory tract infections, Case report

## Abstract

Congenital tracheobronchomegaly, also known as Mounier Kuhn Syndrome (MKS) is a rare respiratory disorder characterized by dilatation of the trachea and bronchi. We report a case of a 28-year-old male of African descent in Zambia, who presented with a history of chronic productive cough and repeated chest infections since childhood. He had been treated numerous times for lower respiratory tract infections, and had received empiric tuberculosis (TB) treatment based on chest radiograph findings, despite negative sputum microscopy and molecular tests for TB. Investigations revealed normal baseline blood results and sputum results. He however, had markedly increased levels of serum immunoglobulin E, and spirometry showed an obstructive pattern with significant post bronchodilator improvement. High-resolution computed tomography scan revealed tracheal dilatation, extensive bilateral bronchiectasis and tracheal and bronchial diverticula. The latter were also seen on bronchoscopy, confirming the diagnosis of Mounier-Kuhn syndrome. The patient was treated with combined inhaled corticosteroids and bronchodilators, as well as chest physiotherapy for mucus clearance, which led to improvement in his symptoms. Our case highlights how in low-resource settings, chronic lung diseases, particularly bronchiectasis, are often clinically and radiologically mistaken for and presumptively treated as TB (or its sequelae). Mounier-Kuhn syndrome, albeit rare, should be considered in the differential diagnosis of patients with recurrent lower respiratory tract infections or bronchiectasis. Multidisciplinary team meetings can help in the diagnosis of rare lung diseases.

## Introduction

Congenital tracheobronchomegaly, also known as Mounier-Kuhn syndrome (MKS) is a rare respiratory disorder characterized by dilatation of the trachea and bronchi. Patients present with respiratory symptoms and/or recurrent respiratory tract infections. There is a clear male predominance (8: 1), and the disease is more common in smokers. Patients usually present after the third decade of life. The etiology remains uncertain, but histologically the disorder is characterized by atrophy of tracheal and bronchial smooth muscle and elastic tissue, associated with diverticula in the trachea and bronchi [1]. Approximately 400 cases of MKS have been described globally [2], with only 3 cases so far described in sub-Saharan Africa to our knowledge [3-5]. We describe the first case of MKS diagnosed in Zambia.

## Patient and observation

A 28-year-old male presented to the pulmonology outpatient department with recurrent respiratory tract infections and a persistent productive cough for 14 years associated with exertional dyspnea, chest pain and an occasional wheeze. He denied having any constitutional symptoms, hemoptysis, paroxysmal nocturnal dyspnea, palpitations, orthopnea or lower limb swelling. Additionally, he denied any rash, joint pains or joint swellings. Review of other symptoms was similarly unremarkable.

His past medical history was significant for being treated for tuberculosis (TB) twice in 2018 and 2019 based on chest radiograph abnormalities. Microscopy for *Mycobacterium tuberculosis* and molecular testing of sputum specimens were negative on both occasions. The family history revealed his mother and a sister having similar histories of long-standing productive cough of unclear aetiology. He worked at a road construction site for the past 4 years with exposure to dust and cement and admitted to a 5-pack year smoking history.

**Clinical findings:** on pulmonary auscultation, there were bilateral squeaky breath sounds with wheezes that were more prominent anteriorly. The rest of his physical examination was unremarkable.

**Diagnostic assessment:** his baseline laboratory investigations (Table 1) revealed a normal full blood count and white cell differential cell count, as well as normal renal function and normal liver enzymes. His serum immunoglobulin E levels were increased at approximately fifteen times the upper limit of normal levels. *Aspergillus fumigatus* serology and alpha-1 antitrypsin levels were both within normal limits. A human immunodeficiency virus (HIV) test was negative. His bronchial washings were sent for bacterial and fungal cultures, as well as testing for acid-fast bacilli and MTB/RIF-GenXpert®. These results were negative.

**Table 1:** laboratory test results

Lab test	Patient result	Reference
**Standard laboratory tests**		
Haemoglobin	14.9 g/dl	13.0 - 17.0
Mean corpuscular volume	90.7 fl	83.0 - 101.0
White cell count	5.13 x 10^9^ l	4.0 - 10.0
Neutophils	2.09 x 10^9^ l	2.0 - 7.0
Lymphocytes	2.07 x 10^9^ l	1.0 - 3.0
Monocytes	0.68 x 10^9^ l	0.2 - 1.0
Eosinophils	0.25 x 10 9 l	0.02 -0.5
Basophils	0.03 x 10^9^ l	0.02 - 0.10
Platelets	290 x 10^9^ l	150 - 410
Urea	3.11 mmol/l	2.8 - 7.1
Creatinine	64.8 umol/l	59.0 - 104.0
Bilirubin (total)	9.9 umol/l	2.0 - 21.0
Alanine transaminase	21.7 U/l	0.0 - 45.0
Aspartate transaminase	24.3 IU/l	0.0 - 35.0
Total protein	89.3 g/l	60.0 - 78.0
Albumin	38.4 g/l	35.0 - 52.0
Serum alpha-1 antitrypsin	138 mg/ dl	90 - 200
**Immunological tests**		
Anti-aspergillus fumigatus IgG	45.70 mg/L	0.0 - 66.45
Serum immunoglobulin IgA	4.54 g/l	0.41 - 3.49
Serum immunoglobulin IgG	17.5 g/l	6.5 - 16.0
Serum immunoglobulin IgM	0.6 g/l	0.5 - 3.0
Serum immunoglobulin IgE	1521 IU/L	0.0 - 100.0
**Microbiology testing**		
Alere determine™ HIV-1/2	Negative	
MTB/RIF-GenXpert® (bronchial washings)	Not detected	
Microscopy (bronchial washings)	No acid-fast bacilli	
Culture (bronchial washings)	Normal flora	

**Figure 1:**
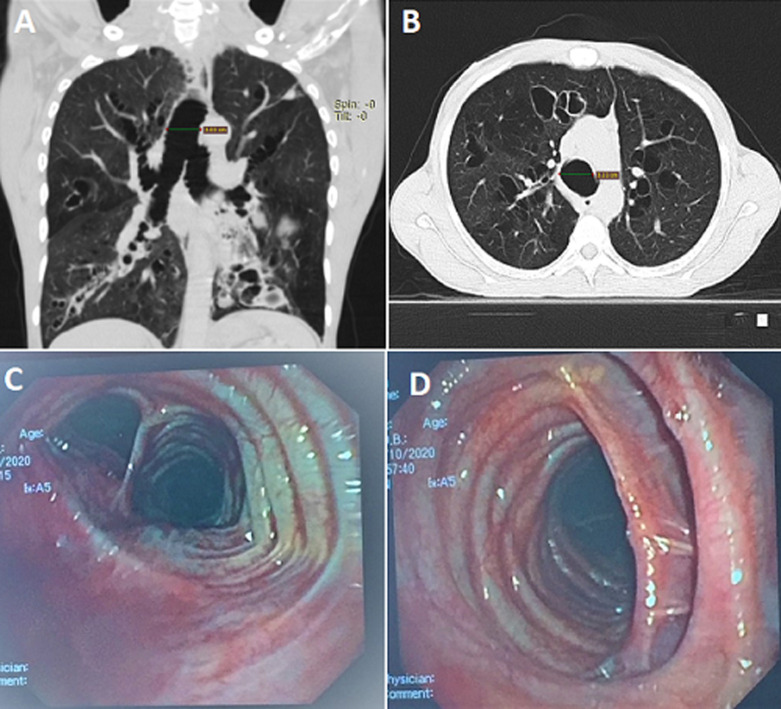
(A,B) chest CT showing a dilated trachea (>3cm), dilated main bronchi, and cystic bronchiectasis, mainly in the lower lobes; C) bronchoscopy image showing a significantly dilated trachea and bronchi; D) tracheal diverticula

The electrocardiogram and echocardiography were normal. Spirometry showed an obstructive pattern with significant bronchodilator responsiveness (Table 2). High-resolution chest computed tomography (CT) revealed a dilated trachea (31mm, measured 2cm above the aortic arch) with diverticula, as well as dilation of the main bronchi (left 24mm and right 21mm) with extensive bilateral cystic bronchiectasis (Figure 1(A,B)). Bronchoscopy revealed normal symmetrical vocal cords with a dilated trachea and main bronchi with multiple diverticula and pouches containing pooled secretions (Figure 1(C,D)).

**Table 2:** pre- and post-bronchodilator spirometry findings

	Pre-bronchodilator	Post-bronchodilator
FEV 1 (L)	1.68	2.02
FVC (L)	2.83	3.52
FEV1/ FVC (L)	59.4%	57.4%

FEV: forced expiratory volume

**Therapeutic intervention:** the patient was started on inhaled fluticasone/formoterol as well as chest physiotherapy. He was also advised on smoking cessation and to switch to an office-based job to limit his exposure to air pollutants.

**Follow-up and outcomes:** the patient reported an improvement in his symptoms and is currently being followed up regularly at the Pulmonology Outpatient Clinic.

**Patient perspective:** “I am glad I finally know the reason for my medical condition. Previously I would just get treated for TB despite all my tests being negative and this was quite frustrating. I now try to avoid things that make my condition worse and live a healthier lifestyle. I now have an office desk job and have quit smoking. In addition, I have started chest physiotherapy and regularly take my prescribed inhaler which has markedly improved my symptoms”.

**Informed consent:** the patient provided written informed consent for this case and the images from his diagnostic tests to be written up and published.

## Discussion

Mounier-Kuhn syndrome (MKS) is characterized by atrophy of tracheal and bronchial smooth muscle and elastic tissue resulting in a markedly dilated trachea and bronchi associated with diverticula. To our knowledge only three other cases of MKS have been reported in sub-Saharan Africa. The first in 1974 in a 28-year-old South-African male [5], the second in a 21-year-old South-African male [4], and the third in a 43-year-old Namibian female [3]. These case reports did not mention any significant exposure to air pollutants. The aetiology of MKS is uncertain, and it not completely clear whether the disease is congenital or acquired. MKS is eight times more common in males. Smokers and those exposed to air pollutants are also at increased risk [1]. MKS has been associated with other connective tissue diseases such as Marfan syndrome, Ehlers-Danlos syndrome, cutis laxa, Ankylosing spondylitis, rheumatoid arthritis, Kenny-Caffey syndrome, and Brachmann-de Lange syndrome [1]. A classification scheme has been proposed that divides patients with MKS into distinct subgroups: following fetoscopic tracheal occlusion or after prolonged intubation, following recurrent pulmonary infections or pulmonary fibrosis, patients with evidence of extra-pulmonary elastolysis, and patients with no clear predisposing factors. The latter group includes most patients described thus far [6]. It has been suggested that MKS is more common among patients of African descent [7]. However, this was not confirmed in a recent literature review of 128 cases of MKS. It was found that approximately 80% of reports did not mention the ethnicity of the patients, and of those who did, the majority were Caucasian [2].

Mounier-Kuhn syndrome can be diagnosed radiologically when high-resolution computed tomography (HRCT) reveals tracheobronchomegaly with diverticula. The airway measurements that can be used to diagnose tracheobronchomegaly are variable, but cutoff values for the trachea diameter of >27mm in males and >23mm in females have been used [1]. The main problems associated with MKS are ineffective cough consequent to pathologic dilation in the tracheobronchial tree and the impairment of mucociliary activity. These cause difficulty in expectorating secretions and lead to recurrent lower respiratory tract infections that ultimately complicate into bronchiectasis, and emphysema. However, patients with no or minimal symptoms have also been reported [1].

As MKS is so rare, the diagnosis is often missed. Limited access to CT-scanners, bronchoscopy and respiratory specialists are also important factors leading to missed diagnosis. Zambia, with a population of over 17 million people has less than 10 CT-scanners and only one respiratory specialist and bronchoscopy unit in the whole country. The diagnosis of this patient was made during a virtual multidisciplinary team meeting. The meeting was set up through a collaboration between the St. Antonius Hospital Interstitial Lung Diseases Centre of Excellence in The Netherlands and the Respiratory Unit at the University Teaching Hospital in Zambia. This highlights the importance of not only multidisciplinary discussions, but also the value of using of virtual means to facilitate mentorship and knowledge transfer.

Additionally, due to the high prevalence of HIV in sub-Saharan Africa, clinicians would be biased towards investigating and empirically treating common HIV-associated respiratory conditions such as TB even in the absence of microbiological confirmation. This was seen in both our case and a Namibian case [3]. In a review of 128 patients with MKS, it was noted that 14 were empirically treated for tuberculosis, despite 50% of these cases yielding negative laboratory TB results [2]. The treatment of MKS is mainly supportive, which includes preventing infections with pneumococcal and Influenza vaccination and treating of infections with antibiotics, along with clearing secretions with mucolytics and chest physiotherapy [1]. In cases where bronchodilator reversibility is present, inhaled corticosteroids and β2-agonist inhalers may also be used. Additionally, surgical treatment involving tracheal stent placement and tracheobronchoplasty has been described with good outcomes [8]. Lung transplantation should be considered in severe cases, but this does present technical difficulties as the grafts need to be connected to the original dilated bronchi. However, successful lung transplant procedures for patients with MKS have been reported [9].

The major anesthetic consideration for patients with MKS undergoing surgical procedures is air leak around the endotracheal cuff resulting in leakage of anesthetic gas and danger of aspiration, tube dislodgement and airway expiratory collapse [10]. Ushakumari *et al*. therefore recommend using the largest diameter endotracheal tube which would pass through the glottic opening and to inflate the cuff so as to prevent air leak and if necessary use wet gauze to reduce further leakage [10].

## Conclusion

Mounier-Kuhn syndrome (MKS) should be considered as a rare cause of bronchiectasis, also in Africa. The presence of dilated trachea and bronchi with diverticula on chest-CT should make one highly suspicious of the diagnosis. Management is usually supportive with avoidance of air pollutants, vaccinations, chest physiotherapy and symptom management and treatment of infections. Our case illustrates the typical delay in diagnosis, as well as empiric TB treatment based on chest X-ray that usually occurs in patients with bronchiectasis despite negative microbiological confirmation of TB. This case highlights the importance of sensitization of healthcare workers on differential diagnosis and management of bronchiectasis as well as the value of (virtual access to) multidisciplinary teams in diagnostically challenging cases.

## References

[1] Krustins E, Kravale Z, Buls A (2013). Mounier-Kuhn syndrome or congenital tracheobroncho megaly: a literature review. Respir Med.

[2] Krustins E (2016). Mounier-Kuhn syndrome: a systematic analysis of 128 cases published within last 25 years. Clin Respir J.

[3] Mkandawire MJ, Muramira NM, Mraba N (2020). A curious case of cough: Mounier-Kuhn syndrome in a Namibian female patient. Pan African Medical Journal.

[4] Trollip MJ (2002). Mounier-Kuhn syndrome: a case study. South African J Radiol.

[5] Bass EM (1974). Tracheobronchomegaly: the Mounier-Kuhn syndrome. S Afr Med J.

[6] Payandeh J, McGillivray B, McCauley G, Wilcox P, Swiston JR, Lehman A (2015). A clinical classification scheme for tracheobroncho megaly (Mounier-Kuhn Syndrome). Lung.

[7] Bateson EM, Woo-Ming M (1973). Tracheobroncho megaly. Clin Radiol.

[8] Odell DD, Shah A, Gangadharan SP, Majid A, Michaud G, Herth F (2011). Airway stenting and tracheobronchoplasty improve respiratory symptoms in Mounier-Kuhn syndrome. Chest.

[9] Dunne B, Lemaître P, de Perrot M, Chaparro C, Keshavjee S (2020). Tracheobronchoplasty followed by bilateral lung transplantation for Mounier-Kuhn syndrome. JTCVS Tech.

[10] Ushakumari DS, Grewal N, Green M (2012). Mounier-Kuhn syndrome: anesthetic experience. Case Rep Anesthesiol.

